# Dentin sialoprotein acts as an angiogenic factor through association with the membrane receptor endoglin

**DOI:** 10.1016/j.jbc.2025.108279

**Published:** 2025-02-06

**Authors:** Ximin Xu, Jing Fu, Guobin Yang, Zhi Chen, Shuo Chen, Guohua Yuan

**Affiliations:** 1 State Key Laboratory of Oral & Maxillofacial Reconstruction and Regeneration, Key Laboratory of Oral Biomedicine Ministry of Education, Hubei Key Laboratory of Stomatology, School & Hospital of Stomatology, Wuhan University, Wuhan, Hubei, China; 2 Frontier Science Center for Immunology and Metabolism, Wuhan University, Wuhan, China; 3 Hubei Provincial Key Laboratory of Developmentally Originated Disease, Wuhan, Hubei, China; 4 Department of Developmental Dentistry, School of Dentistry, The University of Texas Health Science Center at San Antonio, San Antonio, Texas, United States

**Keywords:** angiogenic factor, dental pulp stem cells, dentin sialoprotein, endoglin, endothelial differentiation

## Abstract

Dentin sialophosphoprotein (DSPP) is highly expressed by odontoblasts, the cell type responsible for dentin formation. DSPP therefore has been extensively studied as a regulator of dentinogenesis. Besides defective dentinogenesis in teeth, *Dspp*-deficient mice also display reduced blood vessels in the transition zone of femurs. However, the exact role and underlying mechanisms of DSPP in the process of blood vessel formation remain enigmatic. Here, we show that dentin sialoprotein (DSP), the NH_2_-terminal cleavage product of DSPP, promotes the migration and capillary-like structure formation of human umbilical vein endothelial cells (HUVECs) as well as the migration and endothelial differentiation of human dental pulp stem cells (DPSCs). Further experiments demonstrate that endoglin (ENG), a membrane receptor associated with angiogenesis, can be co-immunoprecipitated by DSP. Flow cytometry assays show that HUVECs and DPSCs, two cell types with endogenous ENG expression, display obvious binding signals of supplemented DSP protein, but human embryonic kidney 293T (HEK293T) cells, a cell type without endogenous ENG expression, do not. Pretreatment with an anti-ENG antibody or knockdown of *ENG* inhibits the binding of DSP to DPSCs, while ENG overexpression enhances binding signals of DSP to HEK293T cells. Meanwhile, multiple experiments demonstrate that knockdown of *ENG* impairs DSP-induced migration and endothelial differentiation of DPSCs. Therefore, ENG is essential for the angiogenic effects of DSP. Moreover, *Dspp*-deficient mice exhibit defective capillary formation in molars, supporting the positive role of DSP in blood vessel development. Collectively, these findings identify that DSP acts as an angiogenic factor through association with ENG.

Since its discovery, dentin sialophosphoprotein (DSPP) has been extensively studied for its role in dentinogenesis (1, 2, 3, 4, 5). It is highly expressed by odontoblasts, the specific cell type responsible for dentinogenesis. Then it is secreted into the dentin extracellular matrix (ECM) to regulate dentin mineralization (1, 6, 7). Heterogeneous mutations in human *DSPP* have been identified to be associated with hereditary dentin disorders including dentinogenesis imperfecta type II (DGI-II), DGI-III, and dentin dysplasia type II (DD-II) (2, 8). According to recognized mutational hot spots in humans *DSPPs*, mouse models with 5′ or 3′ *Dspp* mutations equivalent to those in humans have been established (3, 4). They both exhibit dentin abnormalities (3, 4). Meanwhile, *Dspp*-deficient mice have been obtained, which exhibit mineralization defects in dentin and share a similar phenotype with human DGI-III (5).

After synthesis, DSPP is immediately cleaved into the NH_2_-terminal dentin sialoprotein (DSP) and the COOH-terminal dentin phosphoprotein (DPP), both of which have greater quantity than the full-length DSPP in dentin ECM (9, 10, 11). The cleavage site in mouse DSPP is Asp^452^ and blocking the cleavage of DSPP disrupts its biological function in dentinogenesis (9, 10, 12, 13). The cleavage of DSPP is therefore considered as a biological activation process and several studies have been focused on the function of DSPP cleavage products in the mineralization of collagen fibers during dentin formation (14, 15, 16, 17). *In vivo* transgenic mouse models and *in vitro* cell culture systems have been used to investigate the function of DSP (15, 18, 19, 20, 21). Remarkably, we have observed that agarose beads coated with DSP fragment protein facilitate the formation and invasion of blood vessels during reparative dentin formation (19). In addition, the expression of DSP in mouse odontoblasts which becomes evident starting from embryonic day (E) 17 is concomitant with the fast invasion and active remodeling of blood vessels in the dental mesenchyme (7, 22, 23). Furthermore, apart from teeth, DSPP is moderately or mildly expressed in other organs and tissues, and *Dspp*-deficient mice exhibit reduced blood vessel formation in the transition zone of femurs (24, 25). Therefore, in addition to playing an important role in dentinogenesis, the abovementioned evidence provides hints that DSP may have angiogenic potential.

DSP as a secreted protein has been reported to act as a ligand for membrane receptors of dental mesenchymal cells (19, 20). It can promote the differentiation of dental mesenchymal cells *via* interacting with Occludin or integrin β6 (19, 20). Our previous work preliminarily identified that endoglin (ENG) might interact with DSP (19). Interestingly, ENG is a membrane receptor essential for embryonic vascular development, and ablation of *Eng* in mice leads to defective vasculature development and fetal lethality at E11.5 (26). ENG is highly expressed in mesenchymal stem cells (MSCs). Dental pulp stem cells (DPSCs), a type of tooth-derived MSCs, are positive for ENG and possess high endothelial differentiation potential (27, 28). However, whether DSP can facilitate the angiogenic process of DPSCs dependent on ENG remains enigmatic.

In this study, we purified recombinant DSP proteins and demonstrated that DSP functions as an angiogenic factor to promote the migration and endothelial differentiation of DPSCs. Mechanistically, DSP interacts with ENG, and ENG is required for the angiogenic role of DSP. Furthermore, we find that *Dspp*-deficient mice display defective blood vessel formation in mouse molars, supporting a positive role of DSP in angiogenesis during tooth development.

## Results

### DSP promotes the migration and capillary-like structure formation of human umbilical vein endothelial cells (HUVECs)

**Figure 1:**
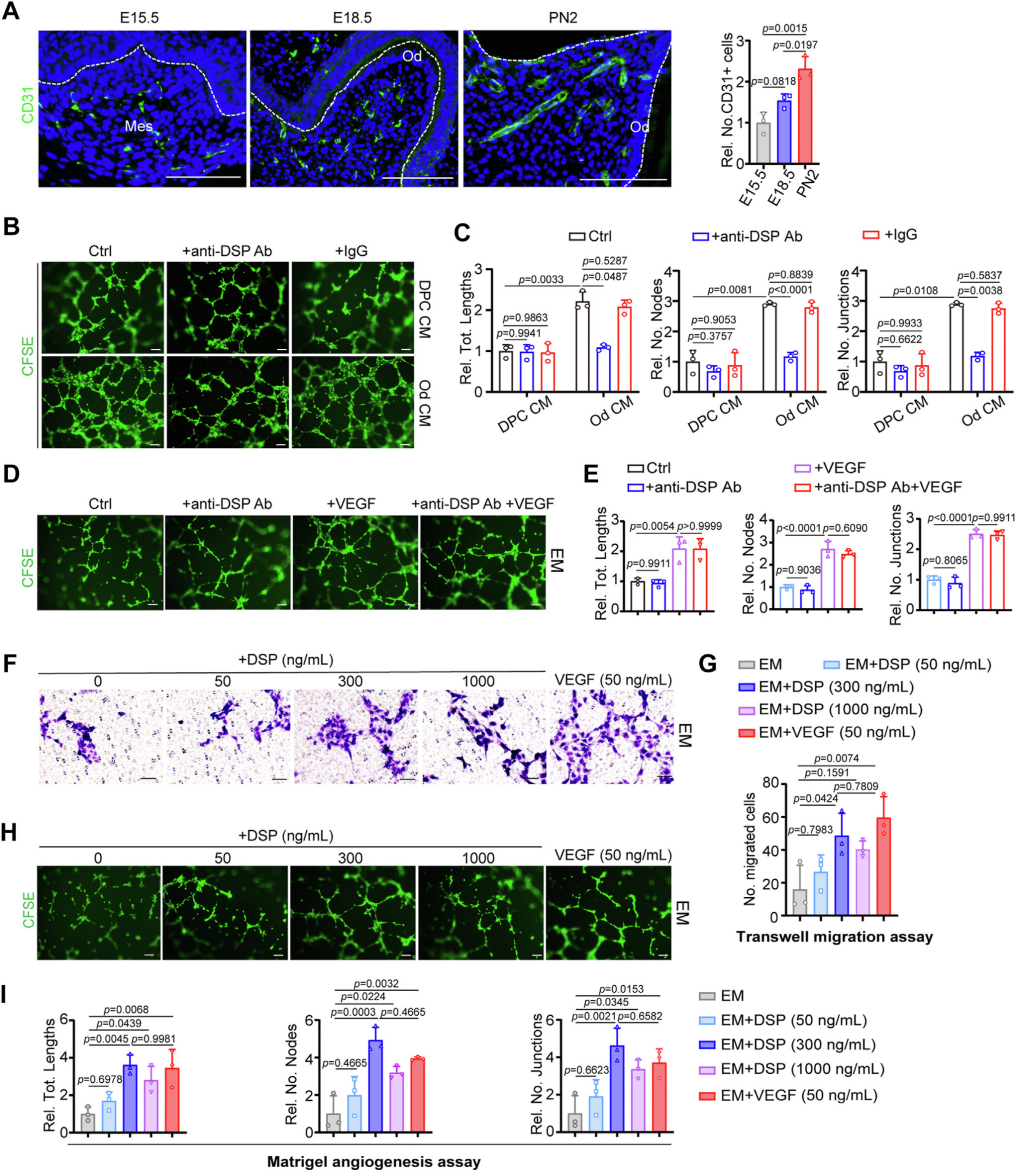
**DSP enhances the migration and capillary-like structure formation of HUVECs.***A*, representative immunofluorescent images of CD31 in murine molars at E15.5, E18.5, and PN2, and quantification of the number of CD31+ cells (n = 3). *B*, representative images of matrigel angiogenesis assays of HUVECs with indicated treatment. HUVECs were cultured on matrigel-coated cell culture plates using indicated conditioned medium (CM) supplemented with or without an anti-DSP antibody (0.5 μg/ml) or isotype IgG (0.5 μg/ml). After incubation for 6 h, cells were stained with CFSE. *C*, quantification of the tube lengths, the number of nodes, and the number of junctions in (*B*) (n = 3). *D*, representative images of matrigel angiogenesis assays of HUVECs with indicated treatment. HUVECs were cultured on matrigel-coated plates using endothelial medium (EM) supplemented with or without an anti-DSP antibody (0.5 μg/ml) and/or VEGF (50 ng/ml). After incubation for 6 h, cells were stained with CFSE. *E*, quantification of the tube lengths, the number of nodes, and the number of junctions in (*D*) (n = 3). *F*, representative images of transwell migration assays of HUVECs incubated with indicated DSP or VEGF for 24 h. Cells were stained with 0.1% (w/v) crystal violet. *G*, quantification of the number of migrated cells in (*F*) (n = 3). *H*, representative images of matrigel angiogenesis assays of HUVECs with indicated treatment. HUVECs were cultured on matrigel-coated cell culture plates using EM supplemented with DSP or VEGF at indicated concentrations. After incubation for 6 h, cells were stained with CFSE. *I*, quantification of the tube lengths, the number of nodes, and the number of junctions in (*H*) (n = 3). The quantification results are represented as mean ± SD (*A*, *C*, *E*, *G*, and *I*). one-way ANOVA with Tukey’s *post hoc* test for (*A*, *E*, *G* and *I*) and two-way ANOVA with Tukey’s *post hoc* test for (*C*). Scale bars: 100 μm for (*A*, *B*, *D* and *H*) and 200 μm for (*F*). Ab, antibody; CFSE, carboxyfluorescein succinimidyl ester; Ctrl, control; DPC, dental papilla cells; E, embryonic day; Mes, mesenchyme; No., number of; Od, odontoblastic cells; PN, postnatal day; Rel., relative; Tot., total.

The vascular system is essential for dentin formation by transporting oxygen and nutrients to odontoblasts (29, 30). To observe the distribution of blood vessels around the onset of dentin formation, immunofluorescent staining of CD31, a marker of endothelial cells (ECs) (31), was performed in mouse molars from E15 to postnatal day 2 (PN2). The results showed that during this period, increased ECs extended from the middle of the dental papilla toward the outer surface of the dental mesenchyme, the odontoblast layer (Fig. 1*A*), implying that odontoblasts may provide a driving force for angiogenesis.

To investigate whether odontoblasts secrete proangiogenic factors, conditioned media (CM) from cultured undifferentiated dental papilla cells (DPCs) or differentiated odontoblastic cells were collected. It has been reported that HUVECs, a commonly used EC model (32), can be induced to migrate and form vasculatures by several angiogenic factors. The collected CM was used to culture HUVECs in Matrigel matrix. Compared with CM from the undifferentiated DPCs, the CM from odontoblastic cells increased the lengths of tubes 2-fold, the number of nodes 3-fold and the number of junctions 3-fold, suggesting that odontoblast secreted factors may have angiogenic potential and thus increased capillary tube formation of HUVECs (Fig. 1, *B* and *C*).

Odontoblasts secrete diverse types of factors, including proteins, exosomes, and metabolites (33, 34). Odontoblasts-derived noncollagenous proteins are essential for dentin mineralization, among which DSP and dentin matrix protein 1 (DMP1) are highly expressed (1, 6). A previous study has verified that DMP1 plays an inhibitory effect on VEGF-induced angiogenesis (35), which excludes DMP1 as a candidate angiogenic factor. To examine whether DSP exhibits angiogenic effect, blocking experiments were performed using an antibody against DSP. In the presence of the anti-DSP antibody, the increased capillary tube formation of HUVECs triggered by odontoblasts-derived CM was not measurably different to that seen with CM form undifferentiated cells (Fig. 1, *B* and *C*). This suggests that DSP in the CM from odontoblastic cells promotes capillary tube formation of HUVECs or that the anti-DSP antibody is inhibitory for angiogenesis. Vascular endothelial growth factor (VEGF) is a representative growth factor with well-known pro-angiogenic activity (36, 37). Further experiments showed that the anti-DSP antibody itself could not suppress capillary tube formation of HUVECs cultured in endothelial medium with or without VEGF supplement (Fig. 1, *D* and *E*). Additionally, Western blot (WB) analysis indicated that the anti-DSP antibody reduced the amount of active DSP available in the CM over the time-course (Fig. S2). Therefore, the above results give a hint that DSP in the odontoblasts-derived CM promotes capillary tube formation of HUVECs.

To confirm the effects of DSP on HUVECs, recombinant DSP protein was synthesized, purified, and identified by WB assays (Fig. S1*A*). Next, HUVECs were treated with DSP at final concentrations ranging from 50 to 1000 ng/ml. VEGF was used as a positive control here, as it has been reported to promote the proliferation, migration, and vascular-like structure formation of ECs (38, 39). Cell counting kit-8 (CCK-8) assays showed that, different from VEGF, DSP does not influence the proliferation of HUVECs (Fig. S1*B*). Remarkably, Transwell migration assays showed that, similar to VEGF, 300 ng/ml DSP increased the number of migrated HUVECs over 2-fold (Fig. 1, *F* and *G*). In addition, Matrigel angiogenesis assays showed that 300 ng/ml DSP increased the lengths of tubes 4-fold, the number of nodes 4.5-fold, and the number of junctions 4.5-fold, which is slightly stronger than the effects of VEGF on the capillary-like structure formation of HUVECs (Fig. 1, *H* and *I*). These results therefore verify that DSP can stimulate the migration and capillary-like structure formation of HUVECs.

Additionally, the concentrations of DSP in the CM were detected using the protein standard curve plotted with the recombinant DSP protein. The results showed that the concentration of DSP was 212 ± 98 ng/ml in the odontoblasts-derived CM and 24 ± 6 ng/ml in the DPCs-derived CM (Fig. S3). Meanwhile, DSP was not seen in the endothelial medium (EM) (Fig. S3).

### DSP induces the migration and endothelial differentiation of DPSCs

**Figure 2:**
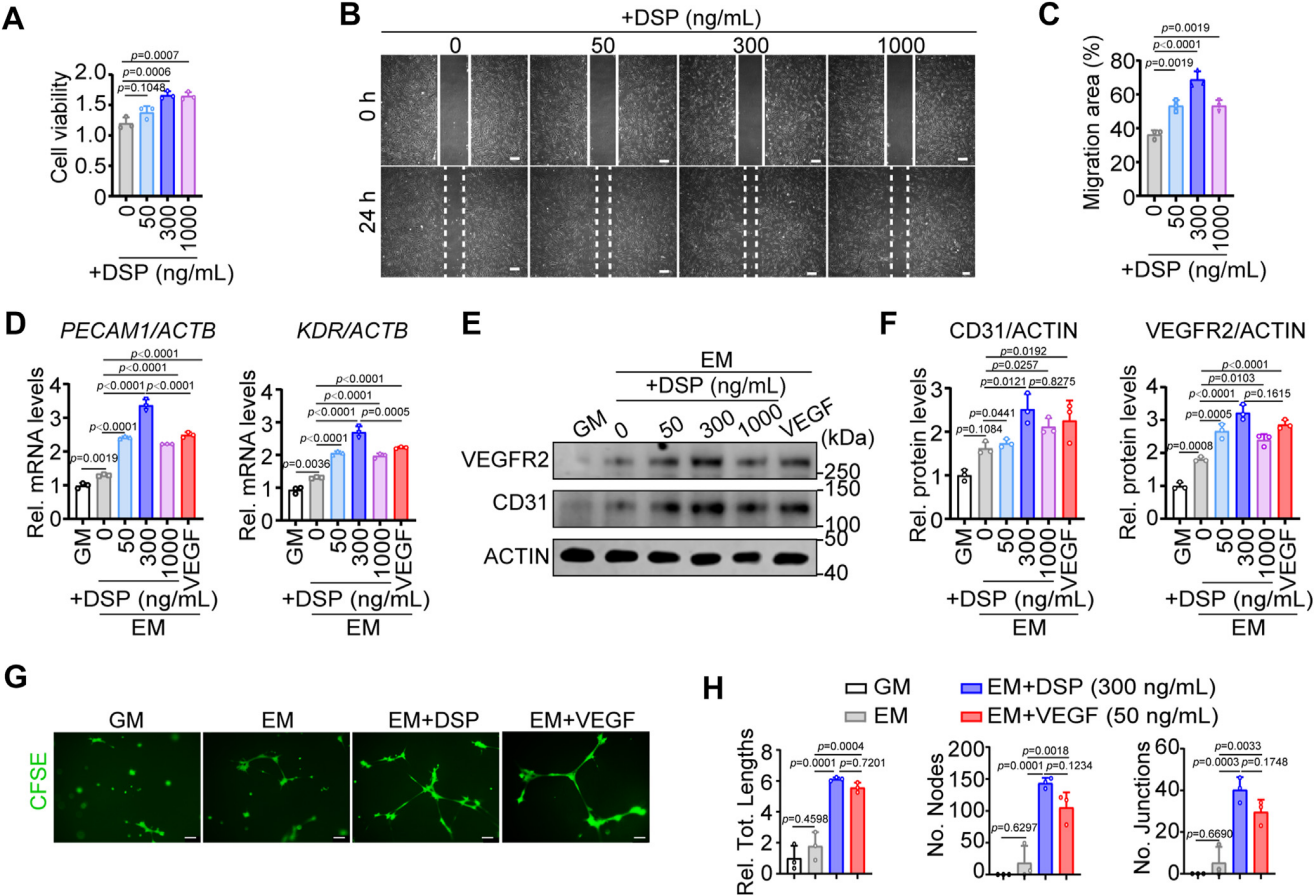
**DSP promotes the migration and endothelial differentiation of DPSCs *in vitro*.***A*, CCK-8 assays show the proliferation of DPSCs after treatment with DSP at indicated concentrations (n = 3). *B*, representative images of scratch wound healing assays of DPSCs after stimulation with indicated DSP at 0 h and 24 h. *Solid lines* and *dashed lines* mark the leading edges of cells at 0 h and 24 h, respectively (n = 3). *C*, quantitative analysis of the migration areas in (*B*). *D*–*F*, DPSCs were cultured in growth medium (GM) or EM supplemented with indicated proteins (VEGF was at the concentration of 50 ng/ml) for 7 days and then collected. The mRNA levels of *PECAM1* and *KDR* of DPSCs with indicated treatment were assessed by RT-qPCR (n = 3). *ACTB*, the gene encoding ACTIN, was used for normalization. The Delta-Delta Ct method was used to analyze data (*D*). The protein levels of CD31 and VEGFR2 in DPSCs with indicated treatment (VEGF was at the concentration of 50 ng/ml) were detected by WB. ACTIN served as a loading control (*E*). Relative densitometric quantitation of (*E*) was performed. ACTIN was used for normalization (*F*) (n = 3). *G*, representative images of matrigel angiogenesis assays of DPSCs. DPSCs were cultured in GM, EM, EM with 300 ng/ml DSP or EM with 50 ng/ml VEGF for 7 days. They were then harvested and seeded onto matrigel-coated plates. After incubation for 14 h, cells were stained with CFSE. *H*, quantification of the tube lengths, the number of nodes, and the number of junctions in (*G*) (n = 3). The quantification results are represented as mean ± SD (*A*, *C*, *D*, *F*, and *H*). one-way ANOVA with Tukey’s *post hoc* test (*A*, *C*, *D*, *F*, and *H*). Scale bars: 200 μm for (*B*) and 100 μm for (*G*).

Among all human tissues and organs, the expression of DSP in teeth is the highest (40). DPSCs are tooth-derived MSCs with multilineage potential (27, 28). Besides odontoblasts, chondrocytes, osteoblasts, and adipocytes, DPSCs can also differentiate into ECs (41), and the migration of DPSCs and their potential to differentiate into ECs are critical for their applications in ischemic diseases. First, we isolated and identified human DPSCs as previously described (21). To observe the effects of DSP on DPSCs, DPSCs were treated with DSP at concentrations ranging from 50 to 1000 ng/ml, and CCK-8 assays as well as scratch wound healing assays were performed. The results of CCK-8 assays demonstrated that DSP at 300 and 1000 ng/ml increased the cell viability of DPSCs (Fig. 2*A*). The migration area of DPSCs with 300 ng/ml DSP treatment increased 2-fold compared to that of control cells, while 50 and 1000 ng/ml DSP increased the migration area 1.5-fold (Fig. 2, *B* and *C*). These results indicate that DSP promotes the proliferation and migration of DPSCs, and 300 ng/ml DSP performs the most obvious effect.

Next, to determine whether DSP was able to influence the endothelial differentiation of DPSCs, DPSCs were cultured in EM supplemented with DSP at different concentrations, while cells cultured in EM with the presence of VEGF (50 ng/ml) and cells cultured in growth medium (GM) were used as positive and negative control, respectively. Real-time quantitative polymerase chain reaction (RT-qPCR) and WB analysis were carried out to detect the expression levels of endothelial cell markers. Platelet and endothelial cell adhesion molecule 1 (*PECAM1*) and kinase insert domain receptor (*KDR*) are genes encoding CD31 and vascular endothelial growth factor receptor 2 (VEGFR2), respectively. The results showed that the mRNA levels of *PECAM1* and *KDR* increased 1.5-fold in DPSCs cultured in EM compared with those cultured in GM (Fig. 2*D*), and the protein levels of CD31 and VEGFR2 increased 1.5-fold and 2-fold, respectively (Fig. 2, *E* and *F*). Treatment with DSP further enhanced the mRNA and protein levels of these endothelial markers and 300 ng/ml DSP exhibited the most obvious effects (Fig. 2, *D*–*F*). Compared with DPSCs cultured in EM, DPSCs cultured in EM supplemented with 300 ng/ml DSP showed 2-fold increase of *PECAM1* and *KDR* mRNAs as well as 1.5-fold increase of CD31 and VEGFR2 proteins. Equivalent changes were found in DPSCs cultured in EM supplemented with VEGF (Fig. 2, *D*–*F*). The concentration of 300 ng/ml was therefore used in the following experiments. Matrigel angiogenesis assays showed that, similar to cells treated by VEGF, DPSCs cultured in EM supplemented with DSP at the final concentration of 300 ng/ml possessed stronger sprouting capability, forming more capillary-like structure than those cultured in GM or EM (Fig. 2, *G* and *H*). The above data indicate that DSP promotes endothelial differentiation of DPSCs *in vitro*.

**Figure 3:**
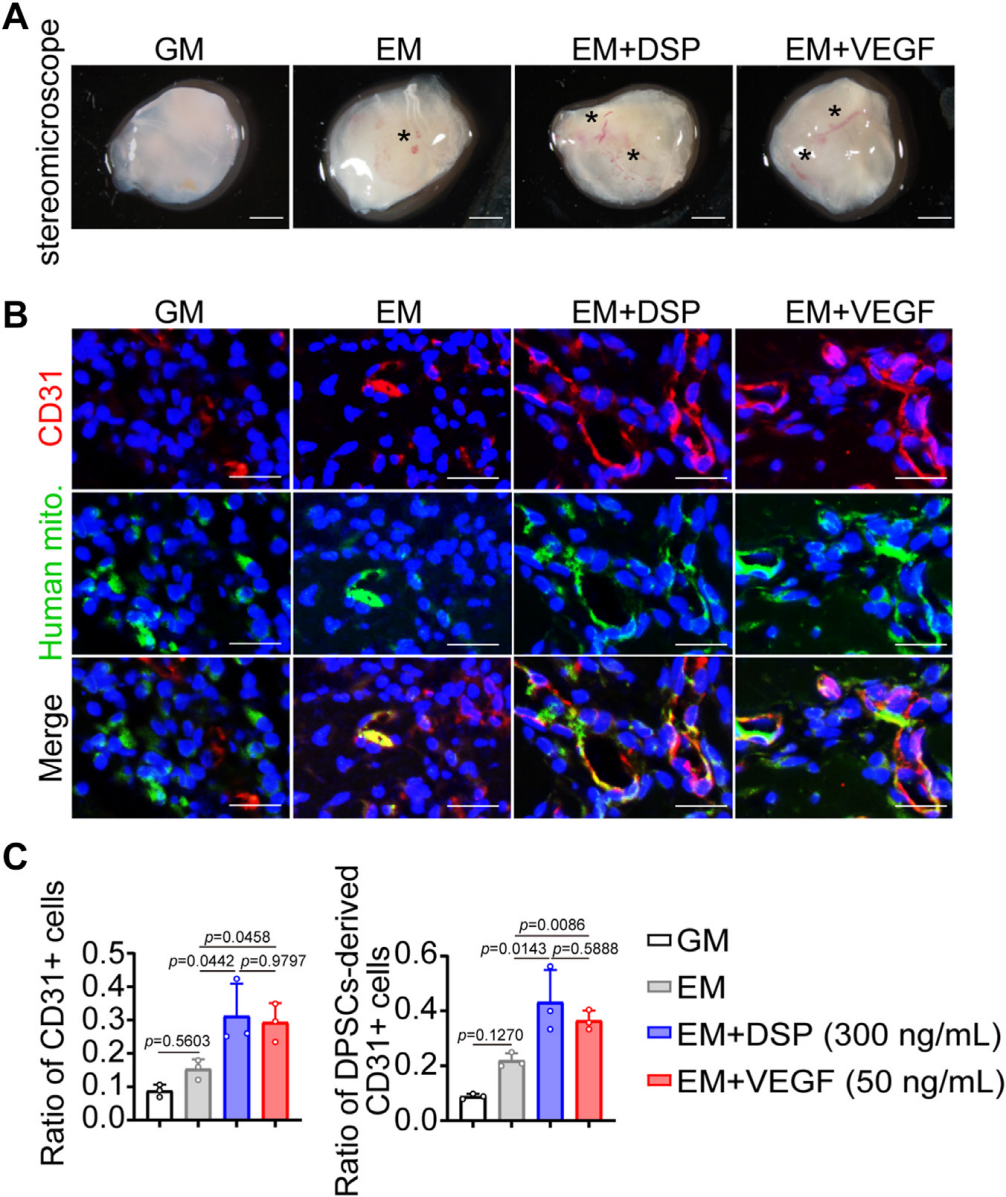
**DSP induces the endothelial differentiation of DPSCs *ex vivo*.** DPSCs were cultured in GM, EM, EM supplemented with 300 ng/ml DSP, or EM supplemented with 50 ng/ml VEGF for 7 days. Then, cells were collected, mixed with matrigel, and injected subcutaneously into the ventral side of mice. After 7 days, the matrigel plugs were harvested. *A*, representative images of matrigel plugs containing DPSCs precultured under indicated conditions. Asterisks mark blood vessels (n = 3). *B*, representative immunofluorescent images of CD31 and human mitochondria in matrigel plugs of (*A*). An anti-human mitochondria antibody was used to mark DPSCs and DPSCs-derived cells. *C*, ratios of CD31+ cells vs. total cells, and DPSCs-derived CD31+ cells vs. total CD31+ cells in (*B*). The quantification results are represented as mean ± SD. one-way ANOVA with Tukey’s *post hoc* test (*C*). Scale bars: 2 mm (*A*) and for 50 μm (*B*). mito., mitochondria; EM, endothelial medium; GM, growth medium.

In an effort to assess whether DSP was able to induce endothelial differentiation of DPSCs *ex vivo*, Matrigel plug assays were performed. Human DPSCs cultured in GM, EM, or EM with the presence of DSP or VEGF were each mixed with Matrigel and injected subcutaneously into BALB/c nude mice. After subcutaneous culture for 7 days, Matrigel plugs containing DPSCs cultured in EM with the presence of DSP showed more blood vessel formation compared with those containing cells cultured in GM or EM (Fig. 3*A*), which was further confirmed by CD31 immunofluorescence (Fig. 3, *B* and *C*). To track the human DPSCs in implanted matrigel plugs, immunofluorescence using an anti-human mitochondria antibody was performed. In the DSP-treated group, the ratio of cells positive for both CD31 and human mitochondria increased 2-fold, suggesting more DSP-treated DPSCs underwent endothelial differentiation (Fig. 3, *B* and *C*). Moreover, DSP-treated cells exhibited similar results to those with VEGF treatment (Fig. 3). Therefore, DSP promotes the endothelial differentiation of DPSCs *ex vivo*.

### DSP is associated with ENG on cell surfaces

**Figure 4:**
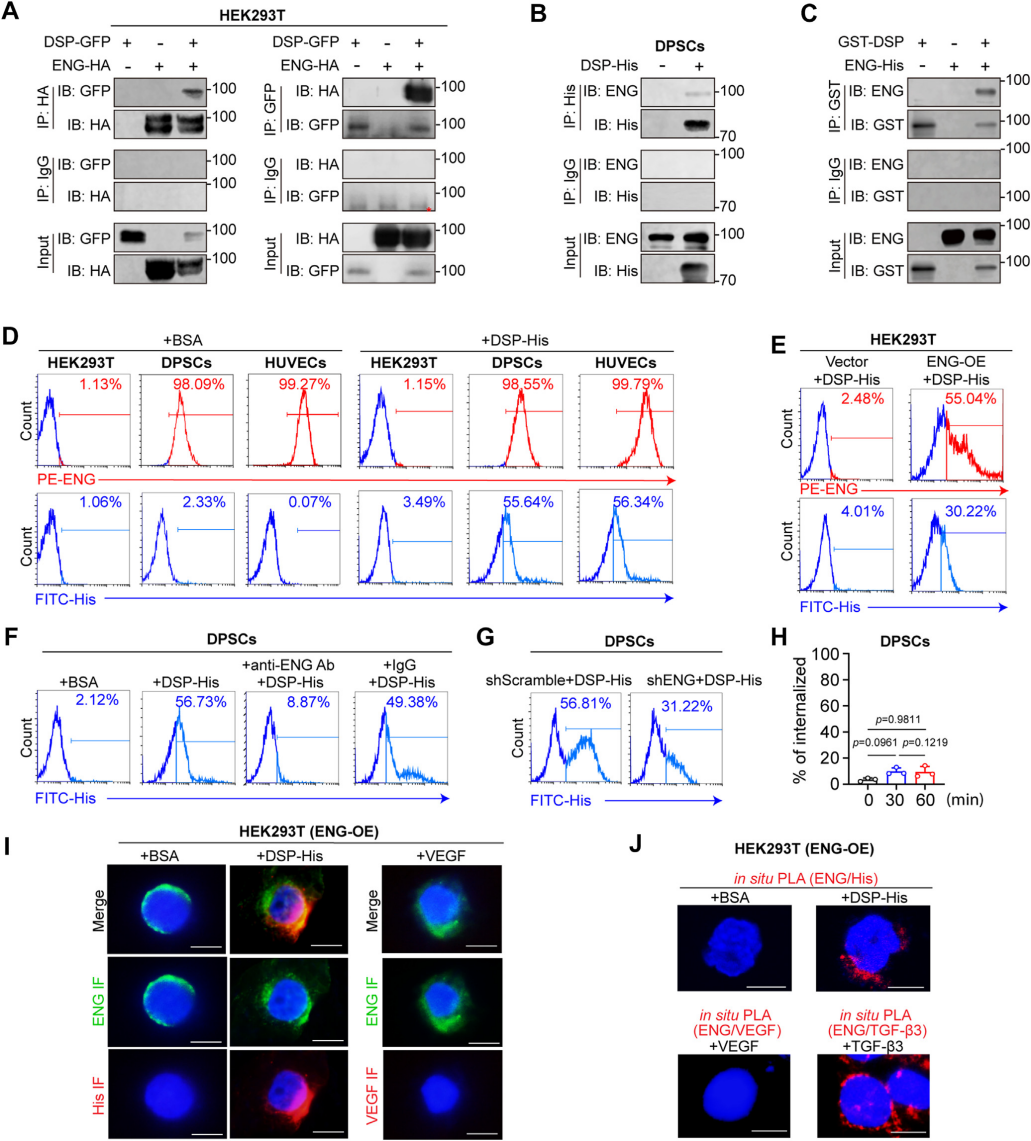
**DSP is associated with ENG on cell surfaces.***A*, co-IP assays using the lysates of HEK293T cells overexpressing DSP and/or ENG demonstrate the association between ectopic DSP and ENG. *Asterisk* (∗) indicates non-specific bands. *B*, the lysates of DPSCs were incubated with purified DSP-His protein. Co-IP assays verified that endogenous ENG in DPSCs was pulled down by exogenous DSP. *C*, *in vitro* incubation of indicated recombinant proteins followed by co-IP assay indicates a direct interaction between GST-DSP and ENG-His. Recombinant His-tagged ENG protein was incubated with recombinant GST-tagged DSP protein and pulled down by an anti-GST antibody, but not by isotype IgG. *D*, HEK293T cells, DPSCs, and HUVECs were incubated with His-tagged DSP or BSA, and flow cytometry analysis were performed using an anti-ENG and an anti-His antibody. *E*, flow cytometry analysis using an anti-His antibody and PE-ENG antibody was performed in HEK293T cells with or without ENG overexpression followed by incubation with His-tagged DSP. *F*, DPSCs were treated with an antibody against ENG or nonimmune IgG followed by incubation with His-tagged DSP or bovine serum albumin (BSA). Flow cytometry analysis using an anti-His antibody was carried out. *G*, DPSCs with or without *ENG* knockdown were incubated with His-tagged DSP. Flow cytometry analysis using an anti-His antibody was carried out. *H*, DPSCs were pretreated with the Rabbit anti-ENG antibody (0.5 μg/ml) at 4 °C for 30 min. After washes, the cells were incubated at 37 °C for 30 or 60 min. Cell surface-bound antibody was detected with Alexa Fluor Red 488 Donkey anti-Rabbit IgG using flow cytometry. The percentages of internalized anti-ENG antibody were then calculated and represented as mean ± SD (one-way ANOVA with Tukey’s *post hoc* test) (n = 3). *I*, representative immunofluorescent images of ENG together with His or VEGF in ENG overexpressed HEK293T cells incubated with His-tagged DSP or VEGF. *J*, *in situ* PLA analysis of ENG together with His, ENG together with VEGF, or ENG together with TGF-β3 in ENG overexpressed HEK293T cells incubated with indicated proteins. Scale bars: 20 μm (*I* and *J*). IF, Immunofluorescence; OE, overexpression.

We next asked which receptor(s) mediates the angiogenic effects of DSP. Beyond as a regulator for dentin mineralization, DSP has been proposed as a ligand and our previous screening experiments suggested a possible interaction of DSP with ENG (19), a membrane receptor crucial for vascular development (26). To confirm their interaction, co-immunoprecipitation (co-IP) assays were performed using lysates of human embryonic kidney 293T (HEK293T) cells with ectopic expression of both ENG and DSP. The results showed that they could pull down each other (Fig. 4*A*). Meanwhile, the lysate of DPSCs was incubated with purified recombinant DSP-His protein. Co-IP experiments showed that endogenous ENG in DPSCs was successfully pulled down by DSP (Fig. 4*B*). Besides, after incubation with recombinant GST-DSP protein, recombinant ENG-His protein was pulled down by an anti-GST antibody, suggesting a direct interaction between ENG and DSP (Fig. 4*C*).

To determine whether DSP was associated with ENG on cell surfaces, first, the relevance between ENG levels and DSP binding signals were analyzed in a panel of cell lines using flow cytometry analysis. The results demonstrated that ENG was highly expressed in HUVECs and DPSCs, but barely detectable in HEK293T cells (Fig. 4*D*). Correspondingly, after incubation with His-tagged DSP protein, HUVECs and DPSCs displayed high signals of DSP-His on cell surfaces but HEK293T cells not (Fig. 4*D*). Meanwhile, when ENG was ectopically expressed in HEK293T cells, the signals of DSP-His increased 7.5-fold (Fig. 4*E*). These results suggest that the level of ENG is positively correlated to the binding of DSP to cell surfaces. Second, DPSCs were pretreated with an anti-ENG antibody or infected with virus containing shRNA targeting *ENG* (shENG) to inhibit the function of ENG. Flow cytometry analysis showed that the binding signals of DSP-His to DPSCs decreased, while pretreatment with a nonimmune IgG or infection with the negative control shScramble virus did not show this effect (Figs. 4, *F* and *G*; S4). Meanwhile, we found that pretreatment with the anti-ENG antibody did not lead to ENG internalization, suggesting that decreased DSP-His signals to DPSCs are due to antibody blocking of ENG (Fig. 4*H*). Third, HEK293T cells with ectopic ENG expression were incubated with BSA, DSP-His, or VEGF followed by double immunofluorescent staining. The results showed co-localization of DSP-His rather than VEGF with ENG on cell surfaces (Fig. 4*I*). Additionally, ENG-overexpressed HEK293T cells incubated with DSP-His showed the duolink proximity ligation (PLA) signals on cell surfaces, while those incubated with VEGF did not (Fig. 4*J*). The cells incubated with TGF-β3, a ligand that shows high affinity with ENG (42), also showed the PLA signals on the surfaces, serving as a positive control (Fig. 4*J*).

### ENG is required for the DSP-triggered migration and endothelial differentiation of DPSCs

**Figure 5:**
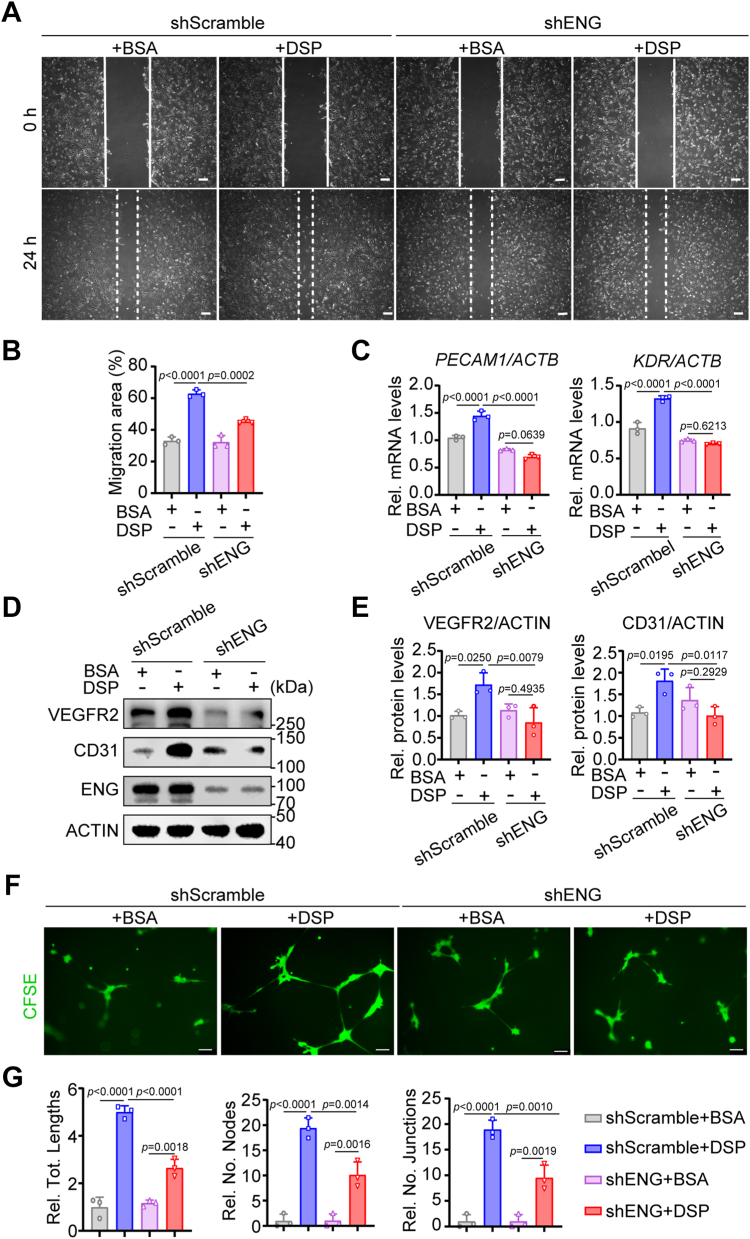
**ENG is required for the DSP-triggered migration and endothelial differentiation of DPSCs *in vitro*.** DPSCs were infected with viruses containing shRNA targeting ENG or scramble shRNA followed by treatment with BSA or DSP (300 ng/ml). *A*, representative images of scratch wound healing assays in DPSCs. The leading edges of cells at 0 h and 24 h after scratch making are marked by *solid* and *dashed lines* respectively. *B*, quantitative analysis of migration areas in (*A*) (n = 3). *C*, the mRNA levels of *PECAM1* and *KDR* in DPSCs (n = 3). The house keeping gene *ACTB* was used for normalization. The calculation method was Delta-Delta Ct. *D*, the protein levels of CD31 and VEGFR2 in DPSCs. ACTIN served as a loading control. *E*, relative densitometric quantitation of (*D*) (n = 3). ACTIN was used for normalization. *F*, representative images of matrigel angiogenesis assays of DPSCs with indicated treatment. After *ENG* knockdown and DSP treatment, DPSCs were then harvested and seeded onto matrigel-coated plates. After incubation for 14 h, cells were stained with CFSE. *G*, quantification of the tube lengths, the number of nodes, and the number of junctions in (*F*) (n = 3). The quantification results are represented as mean ± SD (*B*, *C*, *E*, and *G*). Two-way ANOVA with Tukey’s *post hoc* test (*B*, *C*, *E*, and *G*). Scale bars: 200 μm (*A*), 100 μm (*F*). Rel., relative; No., number of; Tot., total.

**Figure 6:**
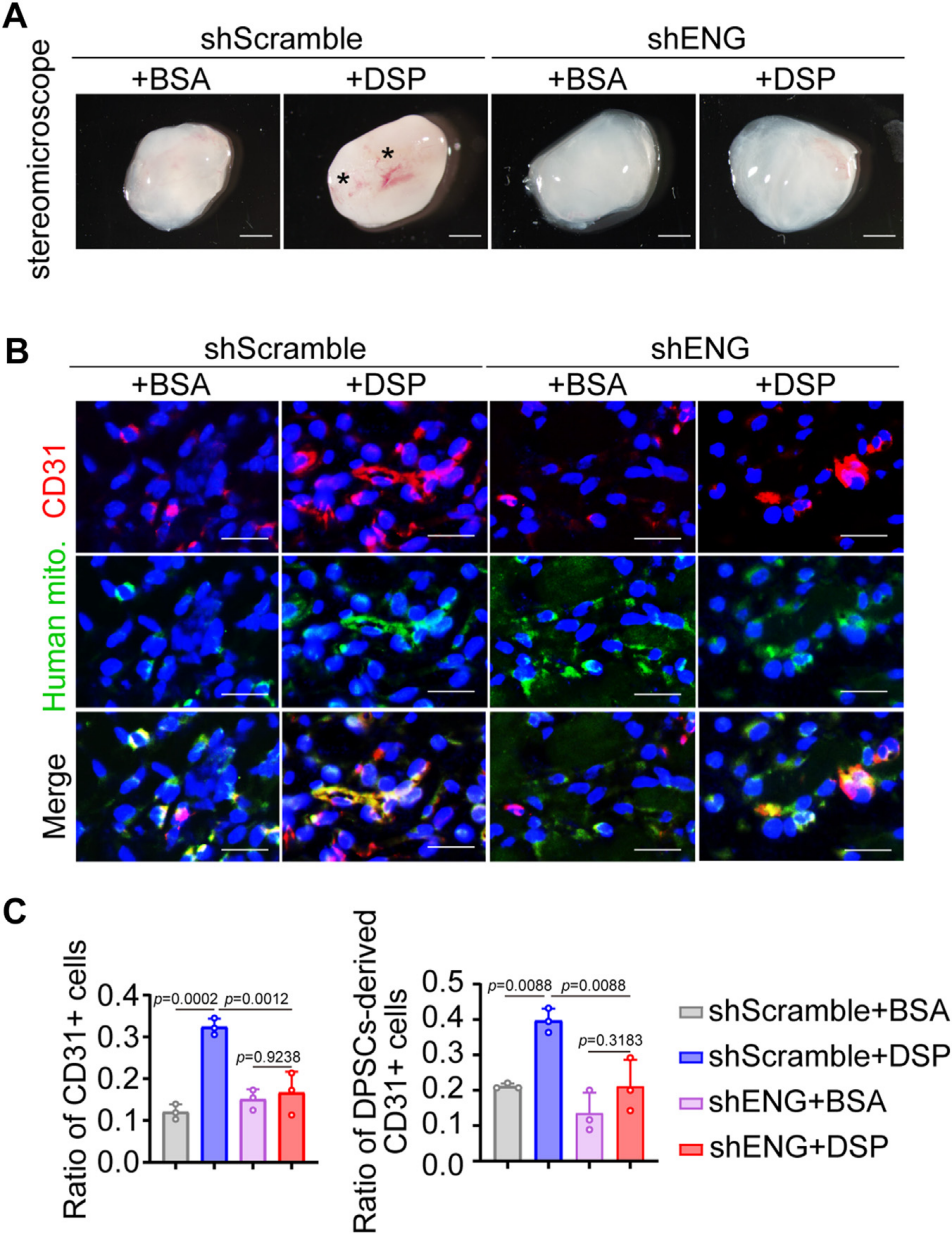
**ENG is required for the DSP-triggered migration and endothelial differentiation of DPSCs *ex vivo*.** DPSCs were infected with viruses containing shRNA targeting ENG or scramble shRNA followed by treatment with matrigel. The mixtures were injected subcutaneously into the ventral side of mice and harvested after 7 days. *A*, representative images of matrigel plugs containing DPSCs with indicated treatment. *Asterisks* show blood vessels (n = 3). *B*, representative immunofluorescent images of CD31 and human mitochondria in matrigel plugs of (*A*). An anti-human mitochondria antibody was used to mark DPSCs and DPSCs-derived cells. *C*, ratios of CD31+ cells vs. total cells and DPSCs-derived CD31+ cells vs. total CD31+ cells in (*B*) (n = 3). The quantification results are represented as mean ± SD (*C*). Two-way ANOVA with Tukey’s *post hoc* test (*C*). Scale bars: 2 mm (*A*), 50 μm (*B*). mito., mitochondria.

To further explore whether ENG mediated the role of DSP in migration and endothelial differentiation of DPSCs, the expression of ENG in DPSCs was knocked down using shENG (Fig. S4). *ENG* knockdown apparently suppressed the DSP-enhanced cell migration as shown by scratch wound healing assays and abrogated the increased expression of *PECAM1*, *KDR*, CD31, and VEGFR2 induced by DSP in DPSCs as shown by RT-qPCR and WB (Fig. 5, *A*–*E*). In addition, *ENG* knockdown also impaired the ability of DSP-treated DPSCs to form capillary-like structure as shown by Matrigel angiogenesis assays (Fig. 5, *F* and *G*). Consistently, Matrigel plug assays showed that increased blood vessel formation contributed by DSP-treated DPSCs was suppressed by *ENG* knockdown in DPSCs (Fig. 6). These results indicate that ENG is required for the DSP-triggered migration and endothelial differentiation of DPSCs.

### Defective vasculature formation in mice deficient of Dspp

**Figure 7:**
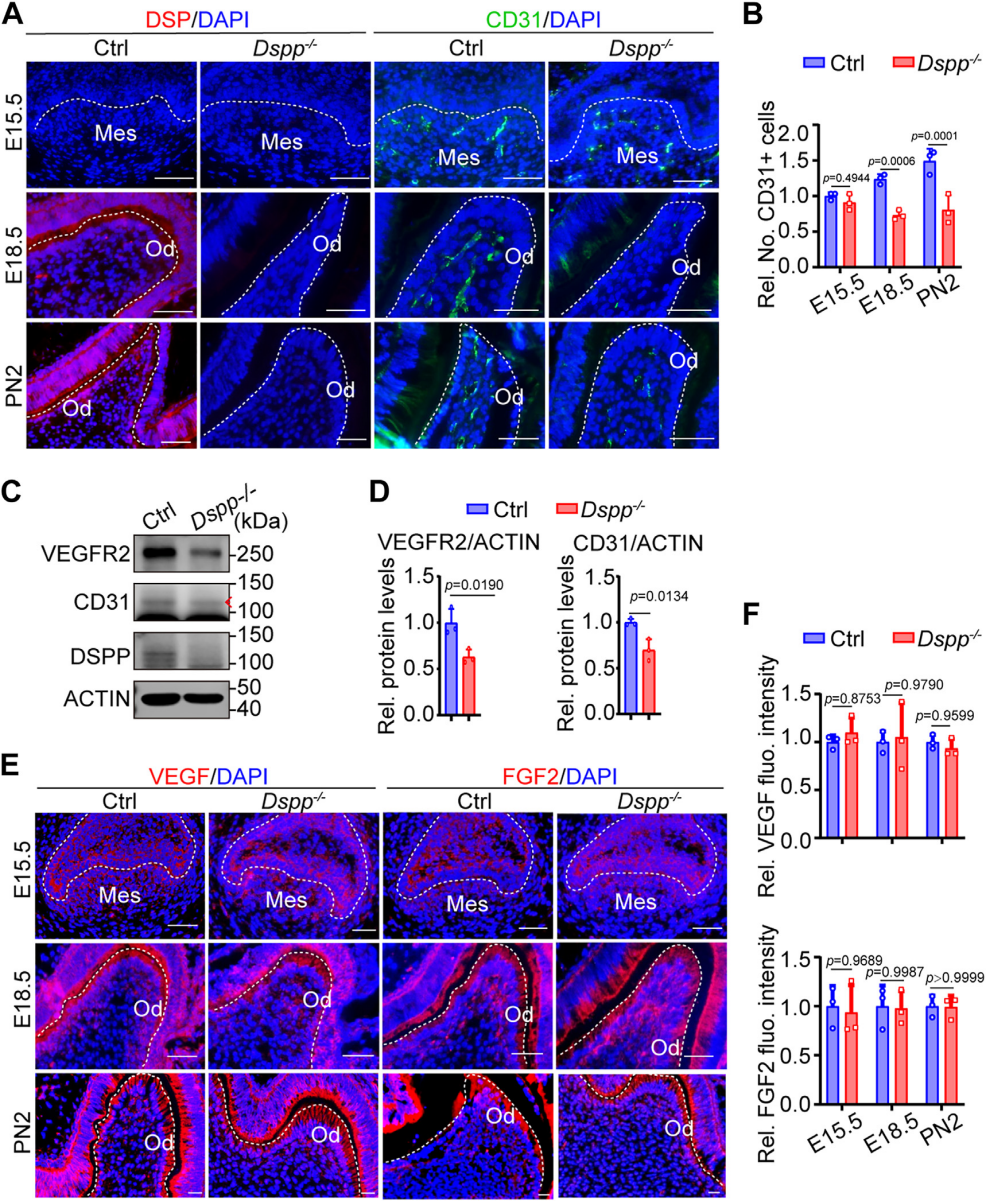
**Defective vascular formation in mice deficient of *Dspp*.***A*, representative immunofluorescent staining of DSP (by using an anti-DSPP antibody targeting the domain of DSP) and CD31 in the molars of wild type (WT) and *Dspp*-deficient mice and their control littermates at E15.5, E18.5, and PN2. *B*, the numbers of CD31+ cells in (*A*) were quantified (n = 3). *C*, the protein levels of VEGFR2, CD31, and DSPP in the dental papilla cells of *Dspp*-deficient mice and their control littermates at PN2 assessed by WB. ACTIN served as a loading control. *Red arrow* indicates the bands of CD31. *D*, relative densitometric quantitation of (*C*) (n = 3). ACTIN was used for normalization. *E*, representative immunofluorescent images of VEGF and FGF2 in the molars of control and *Dspp*-deficient mice at E15.5, E18.5, and PN2. *F*, the mean fluorescence intensity of VEGF and FGF2 in (*E*) was quantified by ImageJ software (n = 3). The quantification results are represented as mean ± SD (*B*, *D* and *E*). Student's *t* test (*B*, *D* and *E*). Scale bars: 50 μm (*A* and *E*). Ctrl, control; fluo, fluorescence; Mes, mesenchyme; No., number of; Od, odontoblast; Rel., relative.

To confirm the role of DSP in blood vessel formation *in vivo*, we examined the vasculature formation in the teeth of *Dspp*-deficient mice around the onset of DSP expression. Immunofluorescent staining showed that DSP was not expressed in the dental mesenchyme at E15.5 but highly expressed in the odontoblast layer at E18.5 and PN2 (Fig. 7*A*). The expression of CD31 was used to represent vascular formation. Immunofluorescence of CD31 showed that *Dspp* deletion led to fewer blood vessels in the dental mesenchyme at E18.5 and PN2 (Fig. 7, *A* and *B*). Additionally, the expression levels of CD31 and VEGFR2 were also reduced in *Dspp*-deficient mice as evidenced by WB analysis (Fig. 7, *C* and *D*). These results suggest that DSPP is required for proper vascular formation during dentin formation.

VEGF and fibroblast growth factor 2 (FGF2) are two important angiogenic factors that have been reported to be involved in regulating angiogenesis in teeth (43, 44). To exclude the possibility that VEGF and FGF2 were associated with defective vascular formation in *Dspp*-deficient mice, immunofluorescent staining and WB analysis were performed. The results showed that *Dspp* deficiency did not affect the expression of VEGF and FGF2 (Figs. 7, *E* and *F*; S5). These results demonstrate that DSP regulates vascular formation of teeth in a VEGF- and FGF2- independent way.

### DSP induces the endothelial differentiation of ENG-positive stem cells from other origins

**Figure 8:**
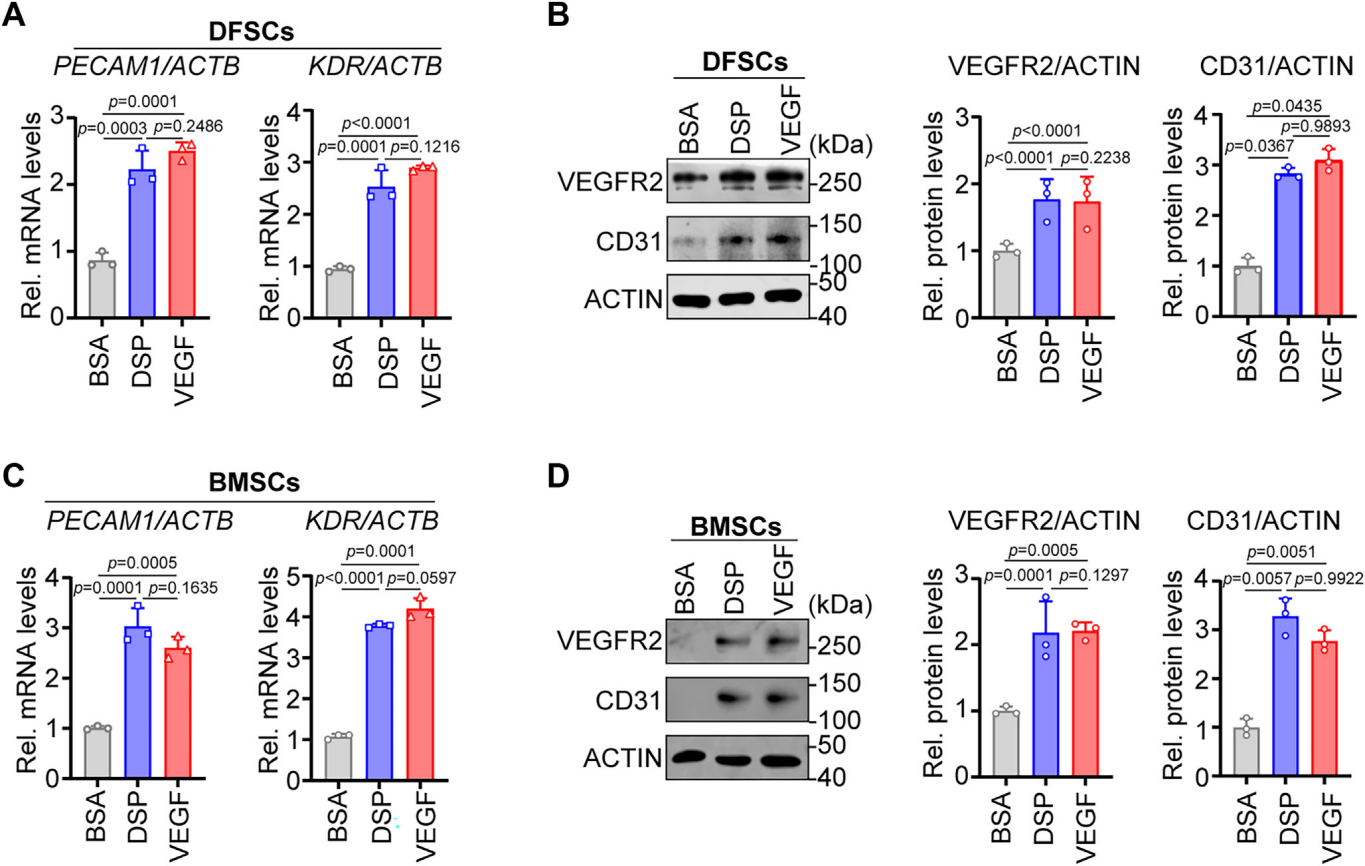
**Endothelial differentiation of DFSCs and BMSCs after treatment with DSP.***A*, the mRNA levels of *PECAM1* and *KDR* in DFSCs treated with BSA, DSP (300 ng/ml), or VEGF (50 ng/ml). *ACTB* was used for normalization. The Delta-Delta Ct method was performed (n = 3). *B*, the protein levels of CD31 and VEGFR2 in DFSCs treated with BSA, DSP (300 ng/ml), or VEGF (50 ng/ml). ACTIN served as a loading control, and relative densitometric quantitation was shown (n = 3). *C*, the mRNA levels of *PECAM1* and *KDR* in BMSCs treated with BSA, DSP (300 ng/ml), or VEGF (50 ng/ml). *ACTB* was used for normalization. The expression was calculated by using Delta-Delta Ct method (n = 3). *D*, the protein levels of CD31 and VEGFR2 in BMSCs treated with BSA, DSP (300 ng/ml), or VEGF (50 ng/ml). ACTIN served as a loading control, and relative densitometric quantitation was shown (n = 3). The quantification results are represented as mean ± SD (*A*–*D*). one-way ANOVA with Tukey’s *post hoc* test (*A*–*D*). Rel., relative.

ENG is widely expressed in many types of MSCs (45). To explore whether DSP promotes the endothelial differentiation of other ENG positive stem cells, dental follicle stem cells (DFSCs) and bone marrow-derived mesenchymal stem cells (BMSCs), two types of MSCs positive for ENG (46, 47) were obtained. Similar to DPSCs, RT-qPCR and WB analysis results demonstrated that both DFSCs and BMSCs showed increased expression of *PECAM1* and *KDR*, as well as elevated levels of CD31 and VEGFR2 after DSP treatment (Fig. 8), suggesting that DSP is able to induce endothelial differentiation of DFSCs and BMSCs.

## Discussion

Over the past years, as a biomarker of differentiated odontoblasts, DSP has been widely considered as a regulator of dentin formation, though its function during dentin formation is still controversial (6, 14, 48). For example, one *in vivo* study showed that transgenic expression of DSP could partly rescue the reduced dentin mineralization of *Dspp*-deficient mice, but another *in vivo* study showed opposite results with severer dentin mineralization defects after transgenic expression of DSP in the *Dspp* null background (15, 18). In the present study, our findings provide the first lines of clear evidence for the angiogenic potential of DSP. We show a previously unreported role of DSP in promoting the migration and endothelial differentiation of DPSCs. Meanwhile, ENG is indispensable for the effects of DSP.

As an organ supplying nutrients and transporting phosphate for dentin formation, vasculature is crucial for dentin formation (22, 29, 30). At the onset of dentin formation, increased blood vessels develop in the dental mesenchyme next to the odontoblast layer (22). We asked whether factors secreted by odontoblasts during dentin formation can influence the angiogenic process. We therefore collected CM from cultured odontoblastic cells, and a positive effect of it on the capillary-like structure formation of HUVECs was found. However, in the presence of an antibody against DSP, the increased vascular network formation caused by odontoblasts-derived CM was suppressed. Subsequently, we demonstrated that DSP was able to promote the migration and capillary-like structure formation of HUVECs, suggesting that DSP might function as an angiogenic factor. DSPP belongs to the Small Integrin-Binding Ligand N-linked Glycoproteins (SIBLING) family. Several members of this family including bone sialoprotein (BSP), osteopontin (OPN), and DMP1 have been reported to participate in regulating angiogenesis (35, 49, 50, 51). Among them, DMP1 impairs VEGF-mediated angiogenesis, while BSP and OPN promote angiogenesis in tumor progression (35, 50, 51). In this study, our data indicate that DSP induces the migration and endothelial differentiation of DPSCs *in vitro* and *ex vivo*.

Considering that DSP is a secreted protein, it is pending to know which membrane receptor mediates the proangiogenic role of DSP. BSP and OPN regulate angiogenesis in an integrin αvβ3-dependent manner (50, 51). The Arg-Gly-Asp (RGD) motif of BSP and OPN has been reported to bind to integrin αvβ3 (50, 51). However, DSP lacks the RGD motif. We previously screened and found that ENG, a receptor critical for blood vessel formation, was a candidate receptor for DSP (19). Co-IP analyses verified that ectopically expressed DSP and ENG were associated with each other, and endogenously expressed ENG could be co-immunoprecipitated by DSP protein. Recombinant DSP protein was able to pull down recombinant ENG, which suggests a direct binding between them. Then, several lines of evidence support that the binding of supplemented DSP proteins to cell surfaces is associated with ENG. First, the expression level of ENG is positively correlated to the DSP binding signals on cell surfaces shown by flow cytometry assays. Second, pretreatment with an anti-ENG antibody or *ENG* knockdown inhibit DSP binding signals on cell surfaces shown by flow cytometry assays. Third, immunofluorescence and *in situ* PLA assays verify that the supplemented DSP protein is co-localized and in proximity with ectopically overexpressed ENG on the surfaces of HEK293T cells.

A previous study has reported that DPSCs positive for ENG show high angiogenic potential (52). In our study, *ENG* knockdown experiments further reveal that ENG mediates the effects of DSP on the migration and endothelial differentiation of DPSCs *in vitro* and *ex vivo*. ENG has been reported as a co-receptor of TGF-β superfamily and is involved in the TGF-β/BMP signaling pathway (42, 53). Additionally, some studies have shown that ENG can function in manners independent of TGF-β (54, 55, 56). However, the exact downstream signaling of DSP-ENG complex needs further investigations.

To further observe the function of DSP in angiogenesis *in vivo*, we obtained *Dspp*-deficient mice. Molars in *Dspp*-deficient mice have been reported to display the phenotype of widened predentin and defective dentin mineralization (5). Here, our data demonstrated that depletion of *Dspp* resulted in a reduction in the number of ECs in teeth, confirming a positive role of DSPP in vascular network formation during tooth development. In addition to the crucial role of DSPP in dentinogenesis, we assume that the phenotype of dentin mineralization defects in *Dspp*-deficient mice may partly be attributed to the reduced transportation of mineralization ingredients due to defective vascular development (29). The expression of other angiogenic factors, VEGF and FGF2, are not affected by the loss of DSPP. Thus, we rule out the possibility that defective vasculature in *Dspp*-deficient mice is indirectly attributed to alterations of VEGF and FGF2 caused by *Dspp* ablation. ENG mediates the regulation of angiogenesis by TGF-β (42). On one hand, *Eng*-deficient mice display defective angiogenesis, and mutations in human *ENG* are associated with a type of vascular disease, hereditary hemorrhagic telangiectasia type-1 (26, 57, 58). On the other hand, continuous ENG overexpression also disrupts physiological angiogenesis in mice (59). Whether activation of ENG to a proper level could rescue the defective vascular formation in *Dspp*-deficient mice needs further investigations.

Collectively, we find that, in addition to enhancing the migration and capillary-like structure formation of HUVECs, DSP also promotes the migration and endothelial differentiation of DPSCs. Mechanistically, DSP interacts with ENG on cell surfaces and knockdown of *ENG* suppresses the DSP-induced migration and endothelial differentiation of DPSCs. Additionally, *Dspp* deficiency causes defective vascular development in mouse molars, supporting the angiogenic role of DSPP. Therefore, in the present study, we expand the role of DSP to an angiogenic factor which requires the presence of ENG. Our previous studies have reported that DSP triggered the odontoblastic differentiation of dental mesenchymal cells *via* other membrane receptors (19, 20). Whether other factors participate in the formation of DSP-ENG complex and are also involved in the DSP-induced migration and endothelial differentiation remains elusive. More investigations and high-throughput experiments, like BIACore, bio-layer interferometry, microscale thermophoresis, and immunoprecipitation mass spectrometry (60, 61, 62, 63), are needed in the future.

## Experimental procedures

### Mice and tissue collection

The animal experiments were approved by the Ethics Committee of the School of Stomatology, Wuhan University (protocol NO. 00279848 and S07924090G) and the Ethics Committee of Center for the Animal Experiment, Wuhan University (protocol NO. WP202110569). Kunming mice were purchased from Hubei Provincial Center for Disease Control and Prevention. Six-week-old BALB/c nude mice were purchased from Hunan Slac Laboratory Animal Co. Ltd. The establishment of *Dspp*-deficient mice in C57BL/6J background was performed in Shulaibao Biotechnology Co., Ltd. The primers for genotyping of mice are listed in Table S1. All mice were housed in ventilated cages in the specific pathogen-free (SPF) facility with a temperature and light regulated.

The mandibles of mice at E15.5, E18.5, and PN2 were isolated and fixed with 4% paraformaldehyde. The tissues were then decalcified, followed by dehydration and paraffin embedding.

### Immunofluorescent staining

Five-μm slices were deparaffinized, rehydrated, and antigen-retrieved. The samples were washed and blocked with 5% BSA. The primary anti-CD31 (1:200, Servicebio), anti-mitochondria (1:800, Abcam), anti-DSPP (1:100, Novus), anti-VEGF (1:100, Abclonal), and anti-FGF2 (1:100, Abclonal) antibodies were used. The secondary antibodies were Alexa Fluor Red 594 Donkey anti-Rabbit IgG (Antgene), Alexa Fluor Red 488 Donkey anti-Goat IgG (Antgene), and Alexa Fluor Red 488 Donkey anti-Mouse IgG (Antgene). Finally, the samples were mounted by DAPI, and images were captured.

### Cell isolation and culture

The research protocols were approved by the Ethics Committee of School of Stomatology, Wuhan University, China (2021-B64), and followed the principles outlined in the Declaration of Helsinki. To isolate tooth-derived cells (DPCs, DPSCs, and DFSCs), the extracted third molars of 18- to 22-year-old patients were collected and washed by phosphate buffered saline (PBS) containing 5% penicillin/streptomycin (P/S) (HyClone) for three times. The sac of the tooth was gently separated from the tooth and the dental pulp tissue was separated from the pulp chamber. The sac and the pulp tissues were each cut into tiny pieces and digested at 37 °C for 1 h. After being filtered and centrifugated, cells were seeded on cell culture flasks and cultured in minimum essential medium alpha (αMEM) (HyClone) supplemented with 10% fetal bovine serum (FBS) (VivaCell SCIENCES) and 1% P/S, which were regarded as passage 0. The cells at passages 3 to 5 were used for the following study. Dental pulp-derived cells were considered DPCs. DPSCs and DFSCs were identified as described in our previous study (64). BMSCs were isolated from the femurs of 5-week-old Kunming mice. After sacrifice, metaphysis in the femur was cut by using sharp scissors, and the bone marrow cavity was washed repeatedly. Bone marrow contents were filtered, centrifugated and seeded. After 5 h, the medium was changed to remove cells that had not adhered. BMSCs were cultured in αMEM (HyClone) supplemented with 10% FBS (Gibco) and 1% P/S. HUVECs were cultured in EM (Sciencell). HEK293T cells were cultured in Dulbecco’s minimum essential medium (DMEM) (HyClone) supplemented with 10% FBS (tbdscience) and 1% P/S (HyClone).

All cells were cultured in a humidified 37 °C incubator with a 5% CO_2_ atmosphere. The medium was changed every other day. To induce the odontoblastic differentiation, DPCs were cultured in the differentiation medium that contains 10 mM sodium β-glycerophosphate (Sigma-Aldrich), 50 mg/ml ascorbic acid (Sigma-Aldrich), and 10 nM dexamethasone (Sigma-Aldrich) for 7 days. For the induction of endothelial differentiation, cells were cultured in the EM for 7 days.

### Conditioned media (CM)

DPCs were induced for odontoblastic cells in differentiation medium for 7 days. Then, the media of undifferentiated DPCs and differentiated odontoblastic cells were replaced with a serum-free medium. After 24 h, conditioned media were collected, centrifuged, and filtered to remove cell debris. The collected media were used in the following experiments.

### Matrigel angiogenesis assay

After pre-cooling at 4 °C, 24-well plates were coated with 200 μl Growth factor–reduced Matrigel matrix (Corning) and incubated at 37 °C for 45 min.

For angiogenesis assays, HUVECs (180,000 cells per well) were cultured on matrigel-coated plates using CM or EM supplemented with the proteins (DSP at 50, 300, 1000 ng/ml or VEGF at 50 ng/ml) or antibodies (0.5 μg/ml anti-DSP antibody or 0.5 μg/ml isotype IgG). After incubation at 37 °C for 6 h, cells were stained with CellTraceTM carboxyfluorescein succinimidyl ester (CFSE) dye (Invitrogen). DPSCs after different treatment were harvested and seeded onto matrigel-coated plates at the density of 180,000 cells per well. After incubation in EM at 37 °C for 14 h, cells were stained with CellTraceTM CFSE dye.

The vascular-like structure was photographed (Olympus Digital Camera). Results were evaluated by ImageJ software. The total lengths, the number of nodes, and the number of junctions were calculated.

### Recombinant protein

To purify recombinant proteins, the pGEX-DSP and pet42b-DSP plasmids were constructed by using ClonExpress II One Step Cloning Kit (Vazyme) according to the manufacturer's instruction and transformed into BL21 (DE3) competent cells (Vazyme). Expression of these proteins was induced with 1 mM IPTG at 25 °C overnight. Recombinant DSP proteins were purified by using Ni NTA Beads (Smart-Lifescience) or GST Agarose (Abclonal) according to the manufacturers’ instructions. Verification of these proteins was performed with Coomassie blue staining and WB assays by using anti-DSP and anti-His antibodies.

Recombinant His-tagged ENG protein and recombinant human VEGF protein were purchased from Abclonal.

### Transwell migration assay and scratch wound healing assay

To evaluate the migratory capacity of HUVECs, 50,000 HUVECs were seeded into the upper chambers Culture Plates (Nest). After adherence, the medium was refreshed to serum-free medium supplemented with DSP (0, 50, 300, 1000 ng/ml) or VEGF (50 ng/ml). After 24 h, the migrated cells were stained with crystal violet (Beytome) and calculated under a microscope (Olympus).

The scratch wound healing assays were performed to evaluate the migratory capacity of DPSCs. Briefly, DPSCs with or without *ENG* knockdown were cultured to confluence. Scratches were made by a 200 μl pipette, and the medium was then changed to serum-free medium containing DSP (0, 50, 300, 1000 ng/ml). Images were captured at 0 and 24 h after scratch-making by a phase contrast microscope (Olympus).

### CCK-8 assay

Cells were seeded into 96-well plates at the density of 1000 cells per well. The medium was changed on the next day and supplemented with recombinant DSP (0, 50, 300, and 1000 ng/ml) or recombinant human VEGF protein (50 ng/ml). Cell Counting Kit (Yeason) was used according to the instructions. After culture for 7 days, the optical density (OD) at 450 nm was detected, and cell viability in each group was calculated.

### RT-qPCR analysis

Total RNAs from cells were collected and reversely transcribed to cDNAs by using the ABScript III RT Master Mix for qPCR with gDNA Remover (Abclonal) according to the manufacturer's instructions. Then real-time quantitative PCR was performed using Hieff qPCR SYBR Green Master Mix (No Rox) (Yeason). The primer sequences are listed in Table S2. *ACTB* was used for normalization. The Delta-Delta Ct method was used for RT-qPCR data analysis.

### WB analysis

The total proteins were harvested by NP-40 (Beyotime) with 1/100 protease inhibitor Cocktail (MedChemExpress) for 10 min at 4 °C and then lysed supersonically. After centrifugation for 10 min with 13,000 rpm at 4 °C, the supernatant was collected, mixed with 5 X SDS-PAGE Protein Sampling Buffer (Biosharp) and denatured at 95 °C for 5 min. Then the protein samples were fractionated by 8%, 10%, or 12% SDS-PAGE and transferred to Trans-blot membranes (Roche). After blocking by 5% skimmed milk for 2 h at room temperature, the membranes were incubated with the primary antibodies at 4 °C overnight as follows: DSPP (NBP2-92546, 1:1000, Novus), VEGFR2 (A11127, 1:1000, Abclonal), CD31 (NBP1-71663, 1:5000, Novus), ACTIN (AC038, 1:8000, Abclonal), GFP (AE011, 1:2000, Abclonal), HA (AE008, 1:5000, Abclonal), ENG (10862-1-AP, 1:1000, Proteintech), His (ab9108, 1:1000, Abcam), GST (10000-0-AP, Proteintech), VEGFA (A12303, Abclonal), and FGF2 (A22448, Abclonal). The membranes were washed with TBST and incubated with a secondary antibody. After washes with TBST, the membranes were observed by WesternBright ECL HRP substrate (Advansta).

### *Ex vivo* matrigel plug assay

After 6-week-old BALB/c nude mice were anesthetized with 2% isoflurane, DPSCs with different treatment were mixed with Ceturegel Matrix Phenol Red-Free, LDEV-Free Matrigel (Yeason), and the mixture in a final volume of 400 μl was injected subcutaneously into the ventral side of mice. After 7 days, the matrigel plugs were harvested for the image capture and following histology analysis.

### Plasmid construction

ENG-HA plasmid was purchased from Sinobiologic. DSP-GFP, GST-DSP, and DSP-His plasmids were constructed by using ClonExpress II One Step Cloning Kit (Vazyme).

### Plasmid transfection and shRNA virus infection

Transfection of plasmids was performed using lipo2000 (Invitrogen) or lipo3000 (Invitrogen) according to the manufacturer’s instructions.

For *ENG* knockdown, viruses containing shRNA targeting *ENG* or scramble shRNA were purchased from Genechem. At around 20% confluence, cells were infected with viral supernatants supplemented with 1 μg/ml polybrene (Yeason). To generate stable cell lines, cells were selected by using 1.5 μg/ml puromycin.

### Co-IP experiments

Cultured cells were rinsed with PBS for three times and lysed using NP-40 (Beyotime) with 1/100 protease inhibitor Cocktail (MedChemExpress) for 10 min at 4 °C. The lysate from HEK293T cells with ENG and DSP overexpression was divided into Input (10%), IP (45%), and IgG (45%) groups. The primary antibodies against HA (Abcloanl, 2 μg) and GFP (Abclonal, 2 μg) or the isotype IgG (Beyotime, 2 μg) were each mixed with the lysate and rotated at 4 °C overnight. The next day, protein A/G magnetic beads (Bimake) were added to the mixture, and the samples were rotated gently for 1 h at room temperature. After washing for five times, the beads were resuspended with 80 μl SDS. The samples were denatured at 95 °C for 10 min and analyzed by WB.

The lysate of DPSCs was mixed with recombinant DSP-His protein (1 μg). After being rotated at 4 °C for 4 h, the mixture was divided into Input (10%), IP (45%), and IgG (45%) groups. The primary antibody against His (Abcam, 2 μg) or the isotype IgG (Beyotime, 2 μg) were each mixed with the mixture and rotated at 4 °C overnight. The following procedures were the same as above.

For co-IP of recombinant proteins, GST-DSP (1 μg) and ENG-His (Abclonal, 1 μg) proteins were mixed and rotated at 4 °C for 4 h. Then the mixture was divided into Input (10%), IP (45%), and IgG (45%) groups. The primary antibody against GST (Proteintech, 2 μg) or the isotype IgG (Beyotime, 2 μg) were each mixed with the mixture and rotated at 4 °C overnight. The following procedures were the same as above.

### Flow cytometry

HEK293T cells with or without ENG overexpression, DPSCs with or without *ENG* knockdown, and HUVECs were incubated with BSA (0.5 μg/ml) or recombinant His-tagged DSP protein (0.5 μg/ml) at 37 °C for 1 h. Then single cell suspensions were collected into the microcentrifuge tubes and washed with cooled PBS (with 2% FBS) three times. The PE anti-human ENG antibody (Biolegend) and anti-His-tag mAb-Alexa Fluor 488 (MBL) were added into 100 μl cell suspensions, and the mixture was then incubated at 37 °C for 30 min. The samples were washed three times again and transferred into FACS tubes. Detection was performed using the CytoFLEX (Beckman Coulter).

### Antibody internalization analysis

DPSCs were incubated with the rabbit anti-ENG antibody (0.5 μg/ml, Proteintech) at 4 °C for 30 min. After washes with PBS containing 2% FBS, cells were incubated at 4 °C or 37 °C for 30 or 60 min to drive possible internalization. Cooled-PBS was used to stopped the internalization reaction and cells were stained with Alexa Fluor Red 488 Donkey anti-Rabbit IgG (Antgene) at 4 °C for 30 min. After washes, the flow cytometry detection was performed using the CytoFLEX. The mean fluorescence intensity that reflects the percentage of internalization of antibody was calculated using the following formula: % internalized = (total surface-bound (4 °C) - total surface-bound (37 °C))/total surface-bound (4 °C) × 100.

### Cell immunofluorescent staining

Cells were fixed with 4% paraformaldehyde, washed with PBS, and blocked with 5% BSA. The rabbit anti-6X His tag (1:100, Abcam), goat anti-ENG (1:40, R&D SYSTEMS), rabbit anti-VEGFA (1:100, Abclonal) were used. The secondary antibodies were Alexa Fluor Red 594 Donkey anti-Rabbit IgG (Antgene) and Alexa Fluor Red 488 Donkey anti-Goat IgG (Antgene). Finally, the samples were mounted by DAPI, and images were captured.

### *In situ* PLA assay

*In situ* PLA was performed by using the Duolink kit (Sigma-Aldrich) according to the instruction. After overexpression of ENG-HA, HEK293T cells were incubated with different recombinant proteins for 1 h at 37 °C. Then cells were fixed, washed, and blocked. The rabbit anti-6X His tag (1:100, Abcam Abcam), goat anti-ENG (1:40, R&D SYSTEMS), rabbit anti-VEGFA (1:100, Abclonal), and rabbit anti-TGF-β3 (1:100, GeneTex) antibodies were used. After incubation at 4 °C overnight, ligation and amplification of PLA probes were performed according to the manual. The samples were mounted with DAPI and the images were captured by fluorescence microscope (Olympus Digital Camera).

### Statistical analyses

The differences between the two groups were analyzed by using Student’s *t* test, and three or more groups were analyzed by one-way or two-way analysis of variance (ANOVA) followed by Tukey’s *post hoc* test. *p* < 0.05 was considered significant. Experiments were repeated at least three times. The results are represented as mean ± SD. GraphPad Prism software (version 8.0) was used to analyze all data statistically.

## Data availability

All data in this study are presented in the article and the supporting information.

## Supporting information

Document Supporting Information contains Figs. S1–S5; Table S1 and S2.

## Conflict of interest

The authors declare that they have no conflicts of interest with the contents of this article.
