# Supplementary material for: Dentin sialoprotein acts as an angiogenic factor through association with the membrane receptor endoglin
Source: J Biol Chem. 2025 Feb 6;301(3):108279. doi: 10.1016/j.jbc.2025.108279 (PMC11910139; doi:10.1016/j.jbc.2025.108279)
Supplement: Supporting information [file mmc1.docx]

**Supporting Information for** **“Dentin sialoprotein acts as an angiogenic factor through association with the membrane receptor endoglin**

**Authors:**

Ximin Xu^1, 2, 3^, Jing Fu^1, 2, 3^, Guobin Yang^1^, Zhi Chen^1^, Shuo Chen^4^, Guohua Yuan^1, 2, 3, *^

**Affiliation:**

^1^State Key Laboratory of Oral & Maxillofacial Reconstruction and Regeneration, Key Laboratory of Oral Biomedicine Ministry of Education, Hubei Key Laboratory of Stomatology, School & Hospital of Stomatology, Wuhan University, Wuhan, Hubei, China;

^2^Frontier Science Center for Immunology and Metabolism, Wuhan University, Wuhan, China;

^3^Hubei Provincial Key Laboratory of Developmentally Originated Disease, Wuhan, Hubei, China;

^4^Department of Developmental Dentistry, School of Dentistry, The University of Texas Health Science Center at San Antonio, San Antonio, TX, United States.

^*^Corresponding author: [yuanguohua@whu.edu.cn](mailto:yuanguohua@whu.edu.cn)

**Keywords:** Dentin sialoprotein, angiogenic factor, endoglin, endothelial differentiation, dental pulp stem cells

Supporting Information document contains Supplementary Figure 1-5 and Supporting Information Table 1-2.


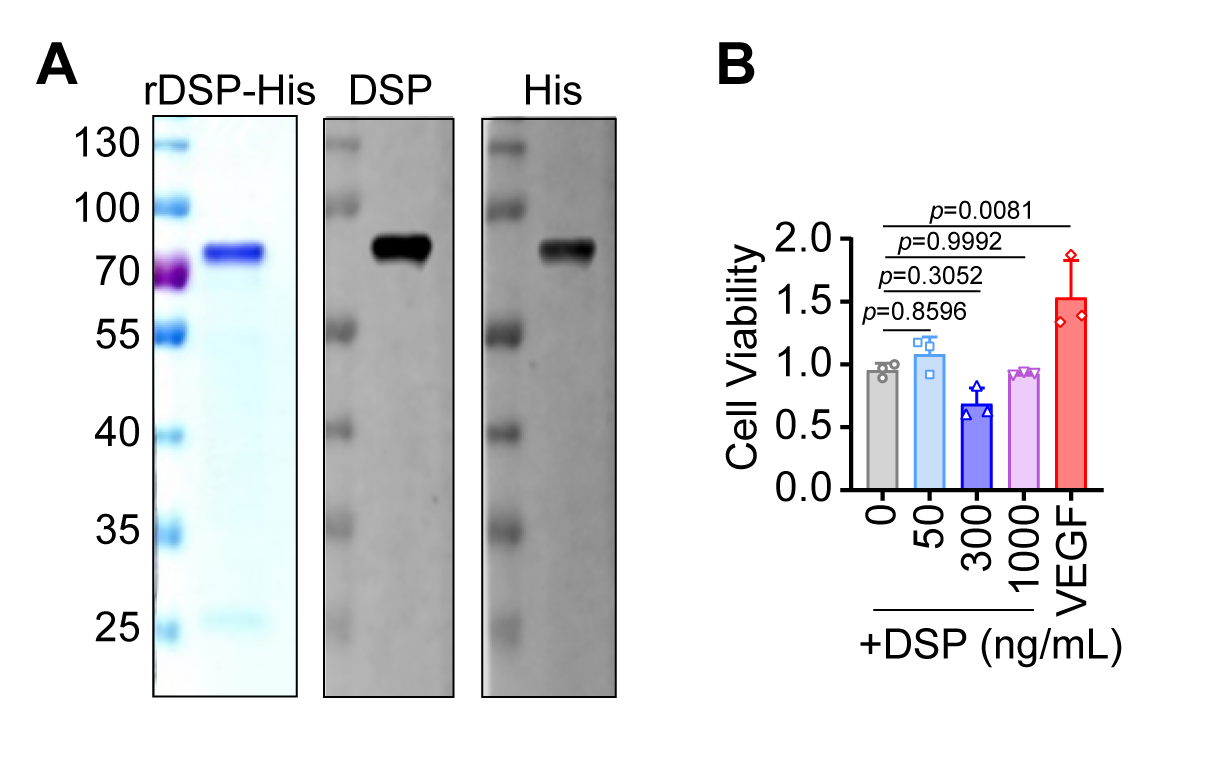
**Supplementary Figure 1.** **DSP does not affect the proliferation of HUVECs.**

*A*, coomassie brilliant blue staining and western blot (WB) analysis of the purified recombinant DSP-His protein.

*B*, CCK-8 assays show the cell proliferation of HUVECs after treatment with DSP or VEGF (n = 3).

The quantification results are represented as mean ± SD (*B*). one-way ANOVA with Tukey’s post hoc test.

**
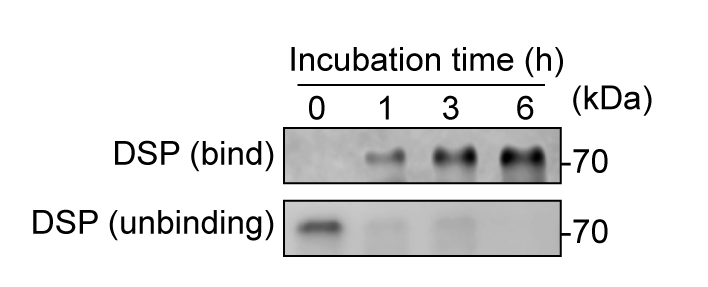
S****upplementary Figure 2. WB analysis indicates the amount of DSP that binds to the anti-DSP antibody and the remnant active DSP in the odontoblasts-derived condition medium (Od CM).** The anti-DSP antibody (0.5 μg/mL) was incubated with Od CM at 37℃ for 0, 1, 3, or 6 h, and then pulled down by protein A/G magnetic beads. The WB analysis demonstrated the amounts of DSP binding with the anti-DSP antibody (the upper blot) and residual active DSP in the Od CM (the lower blot).

**
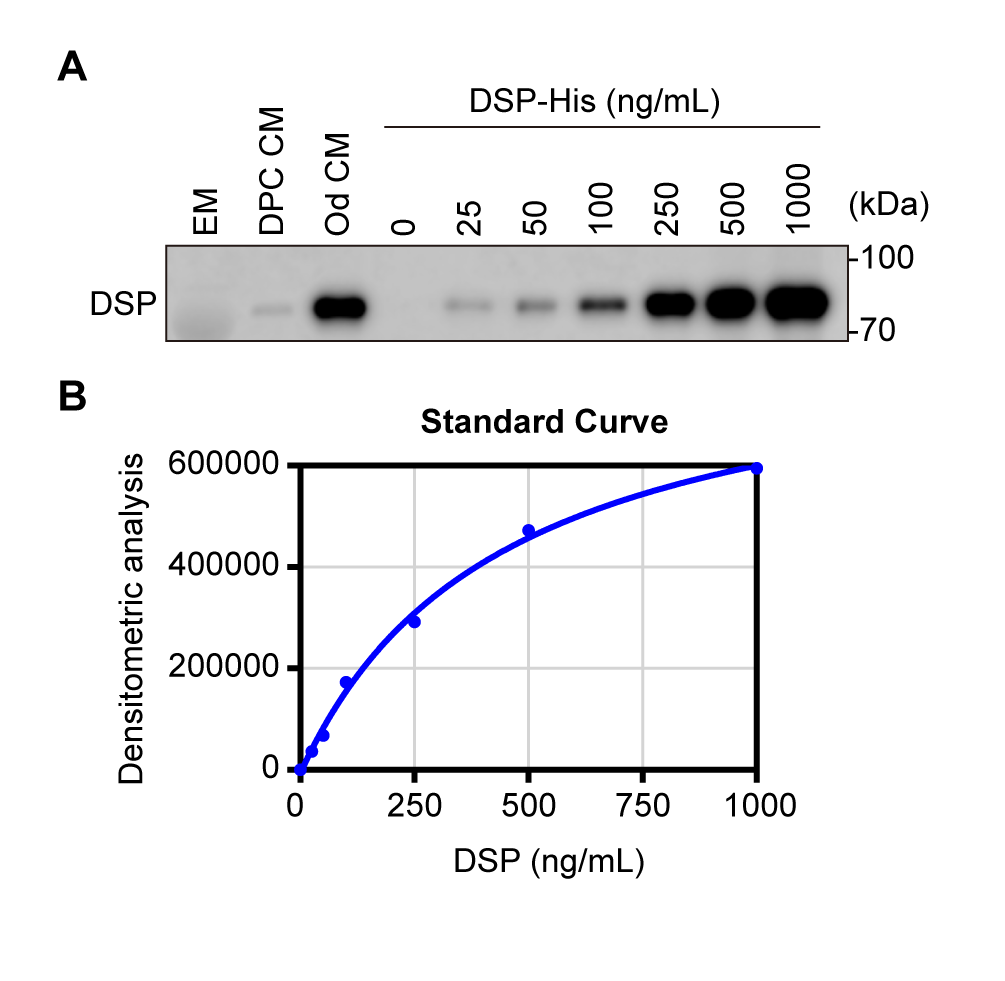
Supplementary** **Figure 3. Detection of the concentrations of DSP in the conditional media (CM).**

*A*, WB assay of the endothelial medium (EM), DPC-derived CM (DPC CM), odontoblasts-derived (Od CM), and purified recombinant DSP-His protein using an anti-DSP antibody.

B, the recombinant DSP-His protein was used to plot the standard curve, which was used to calculate the concentrations of DSP in DPC CM and Od CM.


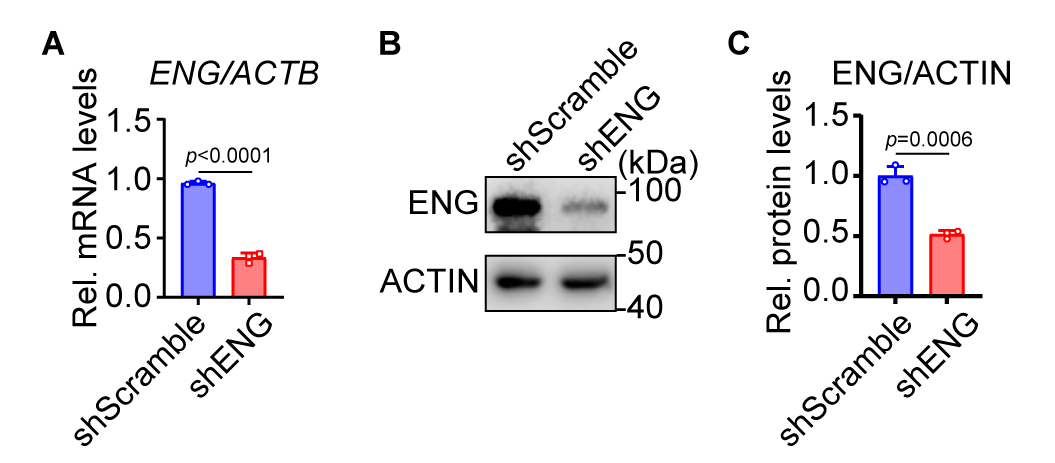
**Supplementary Figure 4. Verification of *ENG* knockdown in DPSCs at mRNA (*A*) and protein (*B* and *C*) levels.** *ACTB*, gene encoding ACTIN, served as a housekeeping gene. The Delta-Delta Ct method was used to analyze data. ACTIN served as a loading control, and the relative densitometric quantification was calculated (n = 3) and represented as mean ± SD (*A* and *C*). Student's t test.


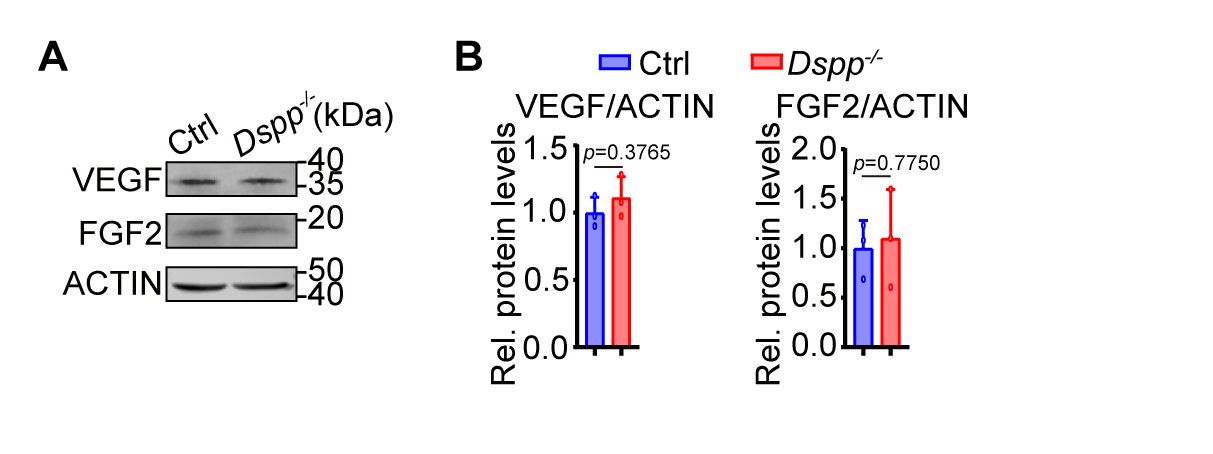
**Supplementary Figure 5. The protein levels of VEGF and FGF2 in the dental papilla cells of *Dspp* deficient and littermate control (Ctrl) mice at PN2.** ACTIN served as a loading control, and relative densitometric quantification was shown (n = 3). The quantification results are represented as mean ± SD (*B*). Student's t test.

**Supporting Information Table 1.** **Sequences of primers used for genotyping of WT and *Dspp* deficient mice.**

| Primer name | Sequence (5’-3’) |
| --- | --- |
| *Dspp*-F1 | TCCCTCAGTCCGAGACTGCATAG |
| *Dspp*-F2 | CTCTATTTGATCCTCAAGTCTCC |
| *Dspp*-R1 | CTCTATTTGATCCTCAAGTCTCC |

**Supporting Information Table 2. The primers used for RT-qPCR.**

| Primer name | Sequence (5’-3’) |
| --- | --- |
| *PECAM1*-F | CCCAGCCCAGGATTTCTTAT |
| *PECAM1*-R | ACCGCAGGATCATTTGAGTT |
| *KDR*-F | GTGATCGGAAATGACACTGGAG |
| *KDR*-R | CATGTTGGTCACTAACAGAAGCA |
| *ACTB*-F | ATTGCCGACAGGATGCAGA |
| *ACTB*-R | GAGTACTTGCGCTCAGGAGGA |
| *ENG*-F | GCATCCTTCGTGGAGCTACC |
| *ENG*-R | GAGGAGTGGTCTGGATCGG |
| *Pecam1*-F | ACCGGGTGCTGTTCTATAAGG |
| *Pecam1*-R | TCACCTCGTACTCAATCGTGG |
| *Kdr*-F | TTTGGCAAATACAACCCTTCAGA |
| *Kdr*-R | GCTCCAGTATCATTTCCAACCA |
| *Actb*-F | GTGACGTTGACATCCGTAAAGA |
| *Actb*-R | GCCGGACTCATCGTACTCC |
